# Intentional Modulation of the Late Positive Potential in Response to Smoking Cues by Cognitive Strategies in Smokers

**DOI:** 10.1371/journal.pone.0027519

**Published:** 2011-11-07

**Authors:** Marianne Littel, Ingmar H. A. Franken

**Affiliations:** Institute of Psychology, Erasmus University Rotterdam, Rotterdam, The Netherlands; University of Granada, Spain

## Abstract

Attentional bias is considered an important concept in addiction since it has been found to correlate with subjective craving and is strongly associated with relapse after periods of abstinence. Hence, investigating in ways to regulate attention for drug cues would be of major clinical relevance. The present study examined deliberate, cognitive modulation of motivated attention for smoking cues in smokers. The effects of three different reappraisal strategies on an electrophysiological measure of attentive processing were investigated. Early and late LPP components in response to passively viewed neutral and smoking pictures were compared with LPPs in response to smoking pictures that were reappraised with three different reappraisal strategies. Results show that when smokers actively imagine how pleasant it would be to smoke (pleasant condition), their early LPP in response to smoking cues increases, but when smokers actively focus on an alternative stimulus (distraction condition) or think of a rational, uninvolved interpretation of the situation (rational condition), smoking-related late LPP amplitude decreases to the processing level of neutral stimuli. Present results are the first to indicate that smoking cue-elicited LPP amplitudes can be modulated by cognitive strategies, suggesting that attentive processing of smoking cues can be intentionally regulated by smokers with various levels of dependence. Although cognitive strategies can lead to enhanced processing of smoking cues, it is not completely clear whether cognitive strategies are also successful in reducing smoking-related motivated attention. Although findings do point in this direction, present study is best considered preliminary and a starting point for other research on this topic. A focus on the distraction strategy is proposed, as there are indications that this strategy is more successful than the rational strategy in decreasing LPP amplitude.

## Introduction

Substance users display cognitive processing biases towards drug-related stimuli [1]. For example, drug users are slower than healthy controls to color name drug-related words on the modified Stroop task [2] and maintain their gaze on drug-related stimuli longer than on neutral stimuli [3]. These biases in cognitive processing are thought to emerge because of the motivational and attention-grabbing properties of drug-related stimuli [4]. According to the incentive-sensitization theory, drug-related stimuli have acquired these properties through repeated drug administration which causes a sensitization of dopamine neurotransmission in the striatum. The ‘incentive salience’ or relevance of stimuli for reinforcement makes the drug-associated stimuli extremely ‘wanted’ and therefore a greater proportion of attentional resources is allocated to them. This drug specific allocation of attentional resources, or attentional bias, is believed to diminish attentional resources left for alternative cues, enhances drug-related cognitions, and causes subjective craving [5].

Enhanced attentional processing of drug cues has indeed been found to be associated to a certain extent with subjective craving in various drug dependent populations [6]. Attentional bias has also been associated with relapse after periods of abstinence, indicating that persons having higher degrees of attentional bias demonstrate higher relapse rates. This relation has been found in smokers [7], alcoholics [8], cocaine [9] and heroin dependent patients [10]. In addition, some recent studies [11], [12] demonstrated that higher levels of attentional bias for alcohol-related stimuli increases the motivation to drink alcohol and that higher levels of attentional bias for smoking stimuli increases the urge to smoke in males [13]. Together, these studies show that attentional bias may play a causal role in addictive behaviours and underline the clinical importance of the concept of attentional bias in alcohol and drug addiction.

Recently, Kober et al. [14] showed that the intensity of subjective craving can be intentionally modulated by cognitive regulation strategies. Smokers and non-smokers were presented with smoking-related and food-related stimuli that are thought to elicit self-reported craving. Participants were instructed to think about the immediate consequence of consuming the pictured substance or the long-term consequences of repeatedly consuming the substance. Reports of craving were reduced when smokers considered the long-term consequences associated with smoking compared to when they considered the immediate consequence of smoking. Furthermore, Kober et al. [15] demonstrated that these craving regulations were supported by the same prefrontal systems of the brain, i.e., the dorsolateral prefrontal cortex, the ventral lateral prefrontal cortex, and the dorsolateral prefrontal cortex – striatal pathways, as the regulation of other appetitive desires (craving for food) and emotion in general.

The regulation of emotion utilizing several different cognitive regulation strategies has been extensively investigated. It has been shown that when participants actively try to reinterpret the meaning of emotional stimuli, autonomic arousal, facial expression, and reports of emotion can be modulated [16], [17], [18], [19], [20]. Furthermore, using fMRI methodology, cognitive regulation strategies have been linked to increased activation of cognitive control regions of the brain, such as the prefrontal cortex, and decreased activation of affective appraisal structures, such as the amygdala [21], [22]. Another common method for studying the effects of cognitive regulation on brain activity has been the use of Event-Related Potentials (ERPs). The later components of the ERP, the P300 and the related Late Positive Potential (LPP), have been found to be enhanced following the presentation of both positive and negative compared to neutral pictures and words [23] and are associated with directed attention toward task-relevant information and facilitated perceptual processing of motivationally relevant stimuli. The P300 component appears to be transient, but the LPP can be enhanced for several seconds after the presentation of emotional stimuli [24]. Because of its sustained duration and its sensitivity to affective properties of pictorial stimuli, the LPP is particularly suited to study the impact of cognitive regulation strategies on emotional responding [23].

Several studies have demonstrated that the amplitude of the LPP is reduced when participants are instructed to decrease emotional responses to negative and positive pictures using self-generated cognitive reappraisal strategies, for example by imagining that the depicted situation gets worse or viewing the pictures from an uninvolved, detached perspective [20], [25], [26]. These LPP reductions were correlated with self-reported changes in emotional intensity. Furthermore, when presenting participants with externally provided reappraisal frames (negative and neutral descriptions prior to the presentation of negative pictures), the magnitude of the LPP is decreased to pictures with neutral reappraisal frames [27] and enhanced to pictures that were preceded by negative instructions [28], [29]. Together, these studies suggest that cognitive regulation strategies, whether self-generated or externally provided, can effectively modulate emotional responding. This applies to self-reported emotion as well as motivated attention, i.e., the activation of attentional and motivational systems of the brain [30], as reflected by LPP magnitude.

ERP studies of substance dependence have demonstrated that the P300 and LPP of substance users relative to healthy controls is enhanced in response to drug-related stimuli compared to neutral stimuli. This result has been obtained in alcoholics [31], [32], [33], heroin users [34], [35], [36], cocaine users [37], [38], [39], cannabis users [40], and smokers [41], [42], [43], [44]. Similar to the view that enhanced P300 an LPP in response to emotional stimuli reflects enhanced motivated attention to these stimuli, it is assumed that the enhancement of the late ERP components in substance users reflects their motivated and elaborate attention for drug-related stimuli. This is underlined by the finding that P300 and LPP amplitudes are significantly correlated with subjective craving [6].

The aim of the present study was to examine whether it is possible to modulate smokers' attentional processing of smoking pictures by deliberate cognitive regulation strategies. Because the enhancement of the late components of the ERP in response to drug cues has been associated with enhanced processing of and motivated attention for smoking cues and because these components, especially the LPP, are sensitive to cognitive regulation strategies in healthy participants, we used LPP amplitude as an outcome measure for successful or unsuccessful regulation of motivated attention for smoking cues in smokers.

To test our hypotheses, we presented the participants with smoking-related stimuli under three instructional conditions. The first instruction was to imagine how pleasant or delicious it would be to smoke the cigarettes depicted in the pictures. The other instructions consisted of a distraction strategy, naming the dominant color in the pictures, and a strategy in which the participants had to view the pictures from an uninvolved perspective, making up a rational story about the content of the pictures. ERPs in response to the reappraised pictures were compared with ERPs in response to passively viewed, non-reappraised smoking pictures and neutral pictures. It was hypothesized that the amplitude of the LPP would be increased during the instruction to think of the pleasant aspects of smoking cigarettes (pleasant condition) and decreased during instructions to focus on other aspects of the smoking pictures or to rationally reinterpret the smoking pictures (distraction and rational condition). Because we expected that, depending on the regulation strategy used, the magnitude of the LPP would change over time [45], we analyzed both the early LPP (600–1000 ms) and the late LPP (1000–2000 ms). There were no specific hypotheses about which regulation strategy would be most successful or how the different LPPs would change over time.

A second aim of the present study was to test whether enhanced attentive processing as reflected by enhanced LPP amplitudes as well as the cognitive modulation of these amplitudes differs between regular smokers and light smokers that do not smoke every day of the week, but at least two days per week for at least two years. Previous studies show that light smokers experience less craving than regular smokers when exposed to smoking cues [14], [46], [47], [48], [49], but are equally distracted by smoking cues when performing a reaction time task [46], and do not differ from regular smokers in the extent to which they can regulate their craving levels in response to smoking cues [14]. It is unknown whether light smokers differ from regular smokers in the electrophysiological processing of smoking cues associated with increased attentional resources and whether they are capable of regulating this attentive processing.

## Methods

### Ethics Statement

The study was conducted in accordance with the Declaration of Helsinki and all procedures were carried out with the adequate understanding and written informed consent of the subjects. The study protocol was approved by the ethics committee of the Institute of Psychology of Erasmus University.

### Participants

Twenty-eight regular tobacco smokers (*mean* age = 21.7, *SD* = 2.57, 39.3% male, 60.7% female) and 22 light smokers (*mean* age = 21.00, *SD* = 1.85, 45.5% male, 54.5% female) participated in the present study. They were recruited at the Erasmus University Rotterdam (the Netherlands) and received either course credit or financial compensation. Smokers were included if they smoked >10 cigarettes per day; light smokers were included if they did not smoke every day of the week, but at least 2 days per week for at least 2 years. Smokers smoked 99.25 cigarettes per week on average (*SD* = 29.08), 14.18 cigarettes per day (*SD* = 4.15, range 10–25), had a mean score of 3.43 (*SD* = 1.83) on the Fagerström Test for Nicotine Dependence (FTND; [50]), indicating low to moderate levels of nicotine dependence, and had a mean carbon monoxide (CO) level of 10.21 parts per million (Ppm; *SD* = 6.96). Light smokers smoked 16.14 cigarettes per week on average (*SD* = 9.87), 5.05 cigarettes per day (*SD*  = 3.02, range 1–13), 3.36 days per week (*SD* = 1.27), and had a mean score of 0.18 (*SD* = 0.50) on the FTND, indicating an absence of nicotine dependence, and a CO level of 2.95 parts per million (*SD* = 2.59). Regular smokers and light smokers significantly differed on FTND score, *t*(48) = 8.05, *p*<0.001 and CO level, *t*(48) = 4.64, *p*<0.001. With regard to smoking duration, regular smokers (*mean* number of years = 5.66, *SD* = 3.58) and light smokers (*mean* number of years = 5.09, *SD* = 1.94) did not show significant differences, *t*(48) = 0.67, *p*>0.10. Furthermore, no group differences were found for age, *t*(48) = 0.88, *p*>0.10, or sex ratio, χ^2^(1) = 0.19, *p*>0.10.

### Self-report measures

Smoking history and demographic data were self-reported (sex, age, smoking duration, number of cigarettes/day, number of days/week). Smoking dependence was assessed with the Dutch version of the Fagerström Test for Nicotine Dependence (FTND) [50]. This questionnaire has good reliability and holds a significant correlation with number of cigarettes smoked per day. The FTND is scored according to the scoring system described in Heatherton et al. [51] and scores range from 0–10. Subjective craving was measured with the brief Questionnaire on Smoking Urges (QSU-brief) [52]. This questionnaire was adapted from the Questionnaire on Smoking Urges (QSU) [53] and consists of two subscales: ‘desire and intention to smoke’ (reward-craving) and ‘reduction of negative affect and withdrawal craving’ (withdrawal-craving). A Dutch translation of the QSU-brief was administered, which has adequate psychometric properties [54].

### Procedure

All participants were instructed to refrain from smoking for at least one hour prior to the experiment in order to avoid direct effects of nicotine on task performance and ERP signals. This was checked at the beginning of the experiment with the EC50 Micro III Smokerlyzer® (Bedfont Scientific, Medford, NJ, USA), a portable device which measures breath carbon monoxide levels (CO Ppm). After providing informed consent, participants filled out questionnaires on demographics, smoking history, subjective craving, and nicotine dependence. After completion, electrodes were attached and the first instructions were given. All participants were tested alone in a sound and light attenuated room. They were all tested by the same experimenter and received the same instructions.

Firstly, the participants were presented with 40 smoking-related and 40 neutral control pictures. They were instructed to watch these pictures closely without employing distracting thoughts. After passive picture viewing the reappraisal part of the task was started. The participants were presented with one of three cognitive reappraisal blocks consisting solely of smoking-related stimuli. They first received a reappraisal instruction and then practiced two pictures with the experimenter. Subsequently, picture presentation was started. After that, the second and the third block were explained, practiced and presented. The order of the three blocks was counterbalanced across participants.

For the pleasant condition, participants were given the following verbatim instructions: *“During this block you will see only smoking-related pictures. You are instructed to imagine how pleasant and delicious it would be to smoke the cigarettes depicted in the pictures or to smoke like the persons in the pictures. Even if you do not like the picture, try to imagine how pleasant and delicious it would be to smoke the presented cigarettes. Hold on to this thought for as long as the picture is presented on the screen. Before each picture presentation you will receive a reminder of the instruction, which in this case will be ‘delicious’.”*

For the distraction condition, participants were given the following verbatim instructions: *“During this block you will see only smoking-related pictures. You are instructed to actively think of the most prominent color in the picture, that is, the color that pops out for you. If no particular color pops out, then pick a color from the picture and keep that in mind. Think of this color for as long as the picture is presented on the screen. Before each picture presentation you will receive a reminder of the instruction, which in this case will be ‘color’.”*

For the rational condition, participants were given the following verbatim instructions: *“During this block you will see only smoking-related pictures. You are instructed to make up a short story about the content of the picture. Think of something that is not directly visible in the picture. For example some background information or something you can easily infer from the picture. The story has to be completely rational, which means that it may not consist of your feelings about the picture, such as ‘this looks nice’. Hold on to the rational thought for as long as the picture is presented on the screen. Before each picture presentation you will receive a reminder of the instruction, which in this case will be ‘rational’.”*

The practice phase consisted of the presentation of two pictures that were the same for all participants. The experimenter asked the participants to respond out loud to the picture according to the reappraisal instruction that was given. After that, the experimenter provided the participants with some alternative options. Most extensively practiced was the rational strategy. Participant responses that included emotions or feelings were strictly corrected (e.g., “she looks pretty” were to be replaced by “she wears make-up because she has a date” or “she probably dyed her hair”). Picture presentation was not started until the experimenter felt that the participants completely understood the instructions.

Each reappraisal block consisted of 40 smoking-related pictures. They were randomly selected from a list of 120 pictures. This list included the smoking-related pictures that were presented in the passive viewing condition. Random selection took place without replacement. This means that there was no overlap between pictures presented in the three reappraisal blocks; there were no pictures that were reappraised with more than one strategy. Pictures were presented for 2000 ms. Prior to each picture, a reminder of the reappraisal strategy appeared on the screen for 1000 ms. Between the reminder and the picture an interval of 500 ms was used. Intertrial interval was 1000 ms.

At the end of the task participants were asked to fill out another craving questionnaire. Then electrodes were removed. E-prime® software (Psychology Software Tools, Pittsburgh, PA, USA) was used for picture presentation.

Neutral stimuli (mean valence level = 5.00, SD = 0.40, range = 4.38−6.21; mean arousal level = 2.65, SD = 0.57, range 1.55–4.71) were selected from the International Affective Picture System (IAPS) [55]. Smoking-related stimuli were downloaded from public online sources and consisted of cigarettes and people holding and smoking cigarettes (mean valence level = 5.97, SD = 0.74, range = 5−8; mean arousal level = 3.30, SD = 1.55, range 1–8). The final stimulus set consisted of 40 neutral pictures and 120 smoking-pictures.

### Electroencephalogram (EEG) recording and signal processing

The electroencephalogram (EEG) was recorded using a digital Active-Two system (BioSemi, Amsterdam, the Netherlands), with active Ag/AgCl electrodes at 34 scalp sites according to the International 10/10 system (32 standard channels mounted in an elastic cap and two mastoid locations, which were used for off-line re-referencing) [56]. The vertical electro-oculogram (VEOG) was recorded with two active Ag/AgCl electrodes located above and underneath the left eye. The horizontal electro-oculogram (HEOG) was recorded with two Ag/AgCl electrodes located at the outer canthus of each eye. An additional active electrode (CMS – common mode sense) and a passive electrode (DRL – driven right leg) were used to comprise a feedback loop for amplifier reference. All signals were digitized with a sampling rate of 512 Hz, a 24-bit A/D conversion, and a low pass filter of 134 Hz. Offline, data were processed with BrainVision Analyzer 2 (Brain products GmbH, Munich, Germany).

The EEG signals were referenced to the mathematically linked mastoids and EEG and EOG were phase-shift-free filtered using a 0.01–35 Hz (24 dB/Octave roll off) band-pass filter. EEG and EOG recordings were segmented in 2100 ms epochs, including 100 ms pre-stimulus baseline. For correction of vertical and horizontal eye movements and eye blinks we applied automatic processing algorithms, i.e., Gratton and Coles algorithm [57]. All ERPs were baseline corrected. Artifact rejection criteria were minimum and maximum baseline-to-peak −75 to +75 µV, and a maximum allowed voltage skip (gradient) of 50 µV for each sample point. Epochs were averaged across trials.

Overall grand averages were obtained for each condition, yielding five conditions: passive-neutral, passive- smoking, reappraisal-pleasant, reappraisal-distraction, and reappraisal-rational. Numbers of artifact-free epochs were respectively 20.36 (*SD* = 9.99), 20.42 (*SD* = 9.46), 20.56 (*SD* = 9.24), 21.34 (*SD* = 9.62), and 20.78 (*SD* = 9.55), and did not differ between stimulus conditions, *F*(4,196) = 0.58, *p*>0.10.

### Analyses

**Figure 1:**
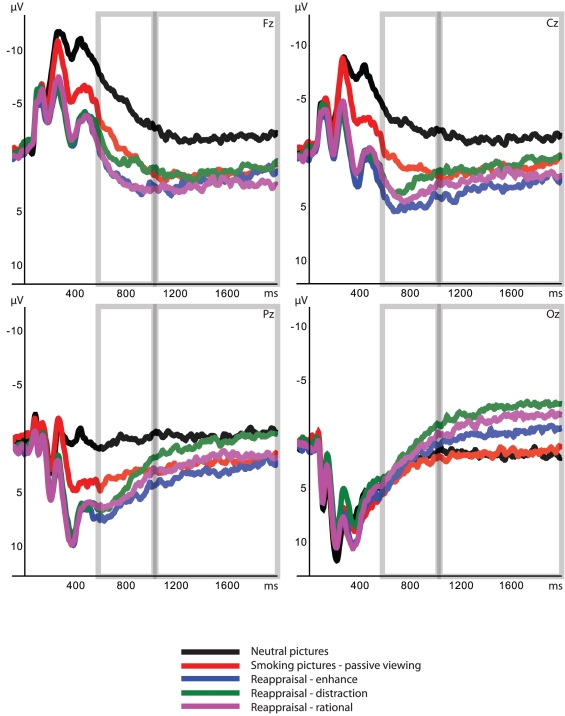
Average event-related potentials (ERPs) in response to the passively viewed neutral and smoking cues and the cognitively reappraised smoking cues for the pooled smokers group at midline electrodes (Fz, Cz, Pz, Oz). Left panels depict the early Late Positive Potential time window (early LPP; 600–1000 ms). Right panels depict the late Late Positive Potential time window (late LPP; 1000–2000 ms).

Visual inspection of resulting ERPs led to the identification of a clear LPP in the 600–2000 ms time frame. Because previous studies have shown that the scalp topography of the LPP shifts around 1000 ms [45] and because a cross-over of waves was visually detected around 1000 ms (see figure 1), the LPP was divided into two components: an early LPP (600–1000 ms) and a late LPP (1000–2000 ms). This was done in order to investigate the attentive processing over time. For both LPP components, mean activities (average amplitude in the time window) were computed per group and stimulus category.

Because the LPP is typically maximal at posterior and parietal electrode sites [23], but group differences in ERP studies of addiction are typically maximal at frontal electrode sites [41], [58], ERP effects were assessed by performing repeated-measurement analyses of variance (ANOVA) on all four midline electrode sites (Fz, Cz, Pz, and Oz), resulting in 4 (electrode)×5 (condition)×2 (group) repeated measures ANOVAs for both the early LPP and the late LPP. To examine exact differences for the significant interaction and main effects, pairwise follow-up analyses with Bonferroni correction were applied to all ANOVAs (Bonferroni corrected p-values are reported). Greenhouse-Geisser correction was applied to all ANOVAs if appropriate (uncorrected *df*'s are reported). Increases in craving between pre- and post measure, i.e., changes in QSU-brief scores after all conditions were administered, were calculated with independent t-tests. An alpha-level of 0.05 was used for all statistical tests.

## Results

### Craving

Regular smokers had a mean score of 3.00 (*SD* = 0.85) on the pre-measure of the QSU-brief, and a mean score of 4.44 (*SD* = 0.84) on the post-measure (after all conditions were administered). This increase reached significance, *t*(26) = 11.70, *p*<0.001, and appeared to be mainly driven by an increase in scores on the QSU-brief subscale ‘reduction of negative affect and withdrawal craving’, *t*(26) = 11.76, *p*<0.001. Light smokers had a mean score of 1.69 (*SD* = 0.74) on the pre-measure, and a mean score of 2.31 (*SD* = 1.07) on the post-measure. This increase was significant, *t*(21) = 4.09, *p*<0.01, and also appeared to be driven by an increase in withdrawal-craving, *t*(21) = 4.86, *p*<0.001. Increases in QSU-brief scores, both for the total QSU-brief and the subscales, were significantly larger for regular smokers than for light smokers, all *t*'s>3.11, all *p*'s<0.01. See table 1 for all mean scores on the QSU-brief and its subscales.

**Table 1:** Mean (SD) craving scores for regular smokers, light smokers, and across all smokers on the QSU-brief and its subscales.

		QSU-Total	QSU-Withdrawal	QSU-Desire
Regular smokers	Pre	3.00 (0.85)	2.97 (0.94)	3.04 (0.87)
	Post	4.44 (0.84)	4.61 (0.89)	4.28 (0.93)
Light smokers	Pre	1.69 (0.74)	1.86 (0.87)	1.52 (0.73)
	Post	2.31 (1.07)	2.59 (1.21)	2.04 (1.00)
All smokers	Pre	2.43 (1.03)	2.48 (1.06)	2.37 (1.11)
	Post	3.49 (1.43)	3.70 (1.45)	3.27 (1.48)

### Electrophysiological data

#### Early LPP

A significant main effect was found for Condition, *F*(4,192) = 12.49, *p*<0.001. See figure 1 (left panels) for average early LPP amplitudes in response to all five conditions. Post-hoc comparisons revealed that all smokers, both the regular and the light smokers, displayed enlarged early LPP amplitudes in response to smoking cues (passive viewing condition) compared to neutral cues, *t*(48) = 5.20, *p*<0.001. Furthermore, early LPP amplitudes were larger for the three reappraisal conditions than for the neutral condition, all *t*'s>4.01, *p*'s<0.01. There were no significant differences between the reappraisal-distraction condition and the passive-smoking condition, or between the reappraisal-rational condition and the passive-smoking condition, both *t*'s<1.40, *p*'s>0.10. However, the difference between the reappraisal-pleasant condition and the passive-smoking condition was significant, *t*(48) = 3.17, *p*<0.05; all smokers displayed more enhanced early LPP amplitudes in response to smoking pictures that were reappraised utilizing the pleasant strategy than in response to smoking pictures that were passively viewed. The main effect for Condition was not moderated by Group (Condition×Group interaction, *F*(4,192) = 0.78, *p*>0.10, Electrode×Condition×Group interaction, *F*(12,576) = 1.06, *p*>0.10), indicating that ERP responding to the five conditions did not differ between regular smokers and light smokers. The main effect for Condition was accompanied by an Electrode×Condition interaction, *F*(12,576) = 10.77, *p*<0.001. At Fz, Cz, and Pz electrodes, LPP amplitudes in response to all smoking stimuli, both passively viewed and reappraised, were more enlarged than the LPP amplitude in response to neutral cues, all *t*'s>3.94, *p*'s<0.01. No effects were found at Oz. The early LPP amplitude in response to the reappraisal-pleasant condition was more enlarged than the early LPP amplitude in response to the passive-smoking condition at both Fz and Cz electrodes, both *t*'s>3.57, *p*'s<0.01. At Fz, the LPP in response to the reappraisal-rational condition was larger than the LPP in response to the passive-smoking condition, *t*(48) = 2.96, *p*<0.05. Furthermore, there was a trend for the LPP in response to the reappraisal-distraction condition to be smaller than the LPP in response to the reappraisal-pleasant condition at this electrode, *t*(48) = 2.71, *p*<0.10. See table 2 for all mean early LPP amplitudes.

**Table 2:** Mean early LPP amplitudes (SD) in microvolt per electrode and condition.

	Fz	Cz	Pz	Oz
Neutral	−5.45 (6.63)	−2.14 (4.80)	0.62 (4.91)	3.35 (4.97)
Smoking	−0.85 (4.39)	1.38 (4.58)	3.90 (3.96)	3.94 (4.80)
Reappraisal – Pleasant	2.39 (5.46)	4.95 (5.45)	5.83 (5.03)	2.91 (5.01)
Reappraisal – Distraction	−0.01 (5.56)	2.99 (5.89)	4.70 (5.98)	2.25 (8.31)
Reappraisal – Rational	1.68 (5.84)	3.65 (5.71)	5.18 (6.18)	1.96 (5.52)

#### Late LPP

A significant main effect of Condition was observed on the late LPP, *F*(4,192) = 3.90, *p*<0.01. See figure 1 (right panels) for average late LPP amplitudes in response to all five conditions. Post-hoc analyses showed that the LPP difference between passively viewed smoking pictures and neutral pictures remained significant, *t*(48) = 3.21, *p*<0.05, as well as the LPP difference between smoking pictures that were reappraised with the pleasant strategy and the neutral pictures, *t*(48) = 3.83, *p*<0.01. Furthermore, the late LPP in response to the passive-smoking condition did not differ from all three reappraisal conditions (passive-smoking versus reappraisal-pleasant, *t*(48) = 0.27, *p*>0.10, passive-smoking versus reappraisal-rational, *t*(48) = 1.16, *p*>0.10), and passive-smoking versus reappraisal-distraction, *t*(48) = 2.57, *p*>0.10). Importantly, the late LPP in response to the reappraisal-distraction condition and the late LPP in response to the reappraisal-rational condition did not differ anymore from the late LPP in response to the neutral condition, both *t*'s<1.50, *p*'s>0.10. This result suggests that after one second the LPP in response to the reappraised smoking cues was reduced to the processing level of neutral cues. There was no significant LPP difference between the reappraisal-pleasant condition and the reappraisal-rational condition, or between the reappraisal-rational condition and the reappraisal-distraction condition, both *t*'s<1.42, *p*'s>0.10. However, the LPP amplitude was significantly smaller for the reappraisal-distraction condition than for the reappraisal-pleasant condition, *t*(48) = 3.07, *p*<0.05. The main effect for Condition was not moderated by Group or Electrode (Condition×Group interaction, *F*(4,192) = 0.68, *p*>0.10; Electrode×Condition×Group interaction, *F*(12,576) = 1.07, *p*>0.10). However, the main effect for Condition was moderated by Electrode, *F*(4,192) = 8.29, *p*<0.001. Post-hoc analyses showed that at both Pz and Oz there were trends for the reappraisal-distraction strategy to elicit smaller LPP amplitudes than the passive-smoking condition, *t*(48) = 2.77, *p*<0.10 and *t*(48) = 2.87, *p*<0.10. At Oz, the late LPP amplitude in response to the reappraisal-distraction strategy was also significantly smaller than the late LPP amplitude in response to the neutral condition, *t*(48) = 4.26, *p*<0.01. Furthermore, at Pz, the LPP amplitude was significantly smaller for the reappraisal-distraction strategy than for the reappraisal-pleasant strategy, *t*(48) = 3.11, *p*<0.05. These results indicate that at occipital-parietal sites the reappraisal-distraction strategy not only reduced LPP responding to the processing level of neutral cues (or beyond), but also reduced LPP responding compared to the passively viewed smoking cues. However, at these sites the difference between LPPs elicited by the passive smoking and neutral cues was not significant, both *t*'s<2.70, *p*'s>0.10. The late LPP elicited by the reappraisal-rational strategy was significantly smaller than the late LPP elicited by the passive-smoking condition at Oz, *t*(48) = 3.54, *p*<0.01, but not at other electrodes. At Fz and Cz there were no significant differences between conditions, except for significant differences between reappraised and passive smoking cues and neutral cues (smoking>neutral). At Cz, however, the reappraisal-distraction condition was the only condition that did not significantly differ from the neutral condition, *t*(48) = 2.29, *p*>0.10. See table 3 for all mean late LPP amplitudes.

**Table 3:** Mean late LPP amplitudes (SD) in microvolt per electrode and condition.

	Fz	Cz	Pz	Oz
Neutral	−2.65 (5.75)	−1.17 (4.94)	−0.48 (6.56)	2.51 (6.69)
Smoking	1.41 (4.15)	1.78 (4.93)	2.35 (4.07)	1.88 (4.83)
Reappraisal – Pleasant	2.29 (4.58)	3.01 (5.37)	2.60 (5.15)	0.08 (5.04)
Reappraisal – Distraction	1.02 (4.63)	1.19 (5.20)	0.32 (5.04)	−1.75 (8.63)
Reappraisal – Rational	1.80 (5.99)	1.75 (6.08)	1.53 (7.04)	−1.49 (5.85)

Note that findings suggest that the distraction strategy is the best strategy to reduce late LPP responding to smoking pictures. However, not all tests yield significant results, probably due to insufficient power. Therefore, an explorative analysis with fewer conditions was performed, namely a 2 (group)×4 (electrode)×3 (condition; passive neutral, passive smoking, reappraisal-distraction) RM ANOVA. As expected, this analysis resulted in a significant main effect for Condition, F(2,96) = 5.51, p<0.01, with the late LPP amplitude in response to distraction strategy being significantly reduced compared to the LPP in response to passively viewing smoking pictures, t(48) = 2.57, p<0.05.

## Discussion

The present study investigated the deliberate, cognitive modulation of attentive processing of smoking cues in smokers as measured with event-related potentials (ERPs). The effects of three different reappraisal strategies on LPP magnitude, an ERP component associated with the allocation of motivated attentional processes [23], were investigated. Early and late LPP components in response to reappraised smoking pictures were compared with early and late LPP components in response to passively viewed neutral and smoking pictures. Furthermore, the present study investigated whether regular smokers differed from light smokers concerning enhanced processing of smoking cues and their ability to modulate this processing by cognitive reappraisal.

### The effects of reappraisal on the electrophysiological processing of smoking cues

Results indicate that early and late LPP amplitudes in response to smoking pictures are differentially modulated by different reappraisal strategies. In the first reappraisal strategy participants were instructed to actively imagine how pleasant and delicious it would be to smoke the depicted cigarettes (pleasant strategy). Employing this strategy resulted in more enhanced LPP amplitudes in response to smoking pictures at fronto-central midline electrodes (Fz, Cz) than employing no strategy (passively viewing) within 600–1000 ms after picture presentation. Because this cue-evoked electrophysiological responding has been associated with enhanced attention toward motivationally relevant stimuli [23], or motivated attention, it can be carefully inferred that when smokers actively imagine how pleasant it would be to smoke, their motivated attention for smoking cues increases. The second reappraisal strategy (a distraction strategy in which participants had to focus on the main color in the picture) and the third reappraisal strategy (a rational strategy in which participants were instructed to make up a short, rational story about the content of the picture) did not significantly alter LPP responding in this time window. In other words, early LPP amplitudes in response to both smoking pictures reappraised with the distraction strategy and the rational strategy were not enhanced or decreased compared to early LPP amplitudes evoked by passively viewed smoking pictures. Therefore, it appears that actively increasing attention for smoking cues is relatively easier than decreasing attention for smoking cues.

However, within 1000–2000 ms after picture presentation, both the distraction and the rational strategy were shown to reduce LPP amplitudes. In contrast to the early timeframe, no significant differences were observed anymore between LPP amplitudes elicited by smoking pictures that were reappraised utilizing the distraction and the rational strategy and LPP amplitudes elicited by passively viewed neutral pictures within the late timeframe. In addition, at occipito-parietal sites, the LPP amplitudes in response to the distraction strategy showed a trend to be decreased as compared to the LPP amplitudes elicited by passively viewed smoking pictures. It must be noted, however, that at occipito-parietal sites there were no significant differences between the passive viewing conditions (no enhanced attentive processing of smoking cues compared to neutral cues) and that across electrodes both strategies did not reduce LPP amplitudes beyond the processing level of passively viewed smoking pictures. Therefore, there exist two different, opposing interpretations and implications of the late LPP results which will be discussed below.

Although the distraction and rational strategies led to a reduction of electrophysiological processing to the level of neutral stimuli, the late LPP amplitudes in response to these strategies were not significantly smaller than the late LPP amplitudes in response to passively viewed smoking pictures. Therefore it could be argued that the strategies did not decrease the attentive processing of smoking cues. This would implicate that it is best to apply no strategy at all. However, the LPP differences between passively viewed smoking pictures and neutral pictures remained significant throughout the entire timeframe (600–2000 ms). This means that without employing a cognitive strategy, the LPP amplitude in response to smoking pictures does not decrease to the same values as the LPP amplitude in response to neutral pictures. The LPPs in response to pictures that were reappraised, on the other hand, are reduced to these values; after one second there is no significant difference between reappraised smoking pictures and neutral pictures anymore. Therefore it could be argued that the strategies did decrease the *enhanced* processing or processing bias (difference in attentive processing between smoking cues and neutral cues) that is normally observed in smokers. This would implicate that cognitive strategies might be useful in reducing attentive processing of smoking-related stimuli.

Although no significant differences were found between late LPP amplitudes in response to pictures reappraised with the distraction strategy and late LPP amplitudes in response to pictures reappraised with the rational strategy, there was a tendency for the distraction strategy to be somewhat more successful in reducing LPP amplitudes than the rational strategy (see also figure 1, right panels). The distraction strategy led to significantly smaller late LPP amplitudes than the pleasant strategy, whereas the rational strategy did not. Furthermore, the LPP amplitudes in response to the distraction strategy showed a trend to be decreased as compared to the LPP amplitudes elicited by passively viewed smoking pictures at both Pz and Oz. For the rational strategy, this finding was only obtained at Oz.

### Limitations and recommendations for future research

Overall, the results suggest that electrophysiological responding to smoking cues can be both enhanced and reduced by intentional, cognitive regulation. However, in contrast to the pleasant strategy, which significantly increased electrophysiological responding to smoking cues compared to passively viewing smoking cues, the rational and distraction strategies did not decrease responding compared to passively viewing smoking cues, at least not across all electrode sites. Therefore it is questionable whether these strategies can modulate motivated attention for smoking cues. As noted above, there are some indications that this is the case, especially for the distraction strategy. However, replication studies need to confirm this finding using a slightly different design which can resolve the interpretational issues encountered in the present study. First of all, we suggest that replication studies increase the presentation duration of the stimuli. As can be seen in figure 1, it might be that the LPP in response to the strategies will eventually (>2000 ms) be reduced beyond the level of passively viewed smoking pictures. Secondly, we suggest that replication studies include repetitions of pictures in both passive conditions. In the present design all reappraisal conditions contained a number of pictures that were already presented in the passive viewing condition. This could have increased overall electrophysiological responding to reappraisal strategies as compared to the passive viewing conditions in which no pictures were repeated. Although no significant early LPP differences were observed between the distraction and rational conditions and passive viewing of smoking cues, suggesting no significant influence of picture overlap, picture overlap could still have modulated LPP amplitudes in the late timeframe causing differences between conditions to fail to reach significance. Finally, we suggest that in replication research the number of conditions should be reduced. Because of its explorative nature, the present study yielded five different conditions, thereby reducing overall power of statistical tests. Results from an additional, explorative analysis on present data showed that when only three conditions were compared (passive neutral, passive smoking, reappraisal-distraction), LPP amplitudes in response to the distraction strategy were significantly reduced as compared to LPP amplitudes elicited by passively viewing smoking cues. This result implies that the distraction strategy might be able to reduce enhanced attentive processing of smoking related cues. Therefore, future studies should further investigate in this specific strategy in relation to drug-related motivated attention.

Furthermore, although a large body of literature suggests that enlarged LPP amplitudes are associated with the allocation of attentional resources to motivationally relevant stimuli [23], [24], [30], [64], inferential caution is warranted. In the present study no behavioral measures of attention were included and self-reported craving was not administered before and after each reappraisal block. Therefore one cannot be certain whether the observed enhanced LPP amplitudes indeed reflect enhanced attentive processing or motivated attention for the presented smoking cues.

Although we measured craving, which was shown to increase during the task, it was beyond the scope of our study to examine the associations between the specific reappraisal strategies and changes in craving levels. It is important that future studies investigate in these relationships in order to shed light on the relationship between (enhanced/modulated) electrophysiological processing and motivated attention as well as to be able to draw conclusions about causality. From the present study, it cannot be inferred whether craving levels were influenced by successfully and unsuccessfully employing reappraisal strategies or whether the capability of employing the reappraisal strategies was influenced by craving. However, there already have been some studies investigating the causal impact of different cognitive regulation strategies on craving. In two studies by Kober et al. [14], [15] it was demonstrated that craving increased as a result of thinking of the direct consequences of smoking a cigarette, but decreased as a result of picturing the long-term consequences of repeatedly smoking. Furthermore, in both smokers and cocaine users it was observed that cognitive regulation strategies decreased activity in the nucleus accumbens and the orbitofrontal cortex which are implicated in craving and respectively process the predictive and motivational value of drug-associated stimuli [15], [65]. These studies adopted strategies in which participants had to think of negative consequences or actively inhibit craving without specific instructions and did not investigate drug-related processing biases. In order to find the most successful reappraisal strategies for reducing both craving and attentional bias, both associated with drug use and relapse, future studies need to shed light on whether the abovementioned strategies can additionally lead to successful reductions in attentional bias and whether distraction can also be successful is reducing craving. Furthermore, future studies should investigate the effectiveness of employing laboratory-studied cognitive regulation strategies in addiction treatment.

### Attentive processing of smoking cues and cognitive reappraisal in light smokers

Present findings show that light smokers and regular smokers do not differ in LPP amplitude magnitude enhancement in response to smoking cues compared to neutral cues or reappraised smoking cues compared to passively viewed smoking and neutral cues. This indicates that regular smokers and light smokers have comparable levels of motivated attention for smoking-related stimuli. This finding is consistent with results from a study by Sayette et al. [46] in which regular smokers and light smokers were equally distracted by smoking-related material while performing a reaction time task. It is also consistent with results from a study by Mogg et al. [59] in which low dependent smokers did not differ from moderately dependent smokers in attentional bias as measured with a dot probe task. Moreover, present results indicate that regular smokers and light smokers do not differ in their ability to intentionally modulate attentive processing of smoking cues, which is in line with observations by Kober et al. [14], who found that light smokers and regular smokers were equally successful at deliberately regulating craving levels in response to smoking stimuli. Despite of the similarities between present and previous results, the finding that very low to non-dependent smokers do not differ from moderately dependent smokers with regard to smoking cue-elicited electrophysiological responding contradicts addiction models of attentional bias and the incentive sensitization theory of addiction [4], [5] in which attentional bias is perceived as an incentive sensitization mechanism that plays an important role in maintaining and exacerbating drug dependence. Following these models one would have predicted that more dependent smokers display more enhanced LPP amplitudes reflecting more attentive processing of smoking cues.

There have been some indications that low dependent or light smokers even show *increased* attentional bias as compared to moderately dependent or heavy smokers [7], [59], [60], [61]. These results have been explained by the ‘incentive-habit’ theory of addiction [62], in which it is hypothesized that when addiction progresses, drug use behaviors become more automatic and consequently the role of incentive motivational processes in the maintenance of drug use becomes less important. In other words, after longer periods of dependence, incentive responding to drug cues decreases; while at the same time habit responding increases [59]. This incentive-habit theory of addiction also provides a conceivable explanation for the absence of ERP differences between the regular smokers and light smokers in the present study. It stands apart that targeting cognitive processing biases in the treatment of smoking addiction remains important, since increased attention for drug cues has been associated with increased relapse rates [10], and both daily smoking and occasional smoking have been associated with higher mortality rates and health risks than non-smoking [63].

### Clinical relevance

There is mounting evidence suggesting that attentional bias is associated with drug use [12], [66] and that its strength predicts relapse risk [7], [8], [9], [10], [67], [68]. Therefore, decrement of attentive processing of drug cues could be an important factor in cessation. There already have been some indications that processing biases can be influenced by using cognitive retraining strategies. For example, Fadardi and Cox [11] showed that the retraining of attentional bias utilizing a modified alcohol Stroop task led to a decreased attentional bias for alcohol stimuli and a reduced alcohol intake for at least three months after retraining. More recently, Schoenmakers et al. [69] showed that a visual probe based attentional bias modification training successfully increased the ability to disengage from alcohol-related cues, and more importantly, that this effect generalized to new, untrained stimuli. With regard to smokers, results have been somewhat inconsistent. Two studies demonstrated positive effects of attentional retraining [13], [70], whereas one study failed to find any effects [71]. All in all, these results are promising and imply that attentional retraining sessions might be valuable in the treatment of addiction. However, these techniques all target attentional bias in an implicit way. To the best of our knowledge, present data are the first to indicate that drug-related attentional processing can also be modulated by explicit cognitive strategies. The use of explicit strategies to regulate processing bias might be complementary to or even advantageous over attentional retraining. For attentional retraining to be successful in clinical practice, the implicitly trained disengagement from drug cues must not only be observed in the laboratory but also last and generalize to new and real-life situations. However, it is exactly the literature on the generalizability that is inconclusive, with the main body of research showing no or limited generalizability [12], [72], [73]. A possible advantage of utilizing explicit strategies might be that one can engage in cognitive reappraisal every time one encounters attention-grabbing drug-related stimuli. Furthermore, cognitive coping strategies, although not explicitly targeting attentional bias, have already been found to reduce craving as well as instances of relapse in clinical practice [74], [75], [76]. Present results are the first to show that the functionality of these coping strategies might work through reductions in attention for drug cues which can be measured on the electrophysiological level and that additionally implementing explicit strategies of processing bias regulation in the treatment of addiction might be of clinical relevance.

### Conclusions

Present study shows that smoking cue-elicited LPP amplitudes can be modulated by cognitive strategies, suggesting that attentive processing of smoking cues can be intentionally regulated. The present findings of LPP modulation fit well within a larger body of work that has been done in the field of emotion research [20], [25], [26], [27], [28], [29]. Within this field it has been repeatedly demonstrated that the LPP is sensitive to interactions between enhanced processing of emotions and cognitive processes comparable to the regulation strategies employed in the present study. Instructions to reappraise or experience emotionally valenced stimuli less or more intensely lead to significantly reduced or enhanced LPP amplitudes which, moreover, are associated with reduced or enhanced self-reported valence ratings of the stimuli. Similar to the finding that people are capable of regulating their attention for motivationally relevant stimuli, smokers might be able to intentionally regulate their attention for stimuli that are motivationally relevant to them, i.e., smoking-related stimuli. The present study is the first to indicate that this is perhaps possible and that the application of cognitive strategies might be valuable in the treatment of addiction. There are clear indications that attention for smoking cues can be enhanced by cognitive strategies. However, it must be noted that it is less clear whether cognitive strategies are also successful in reducing smoking-related motivated attention. Although findings do point in this direction, present study is best considered preliminary and a starting point for other research on this topic. Future studies should specifically investigate in deliberate distraction as a possible strategy to reduce motivated attention for smoking cues, increase presentation times to study later effects of cognitive reappraisal, control for repeated picture presentation and increase statistical power. Furthermore, the present study shows that regular smokers with moderate dependence levels (14 cig/day on average) do not differ from light smokers with very low to absent levels of dependence (5 cig/day, 3 days/week on average) with regard to attention for smoking-related stimuli as measured on the electrophysiological level. This might be explained by smokers' decreased incentive responding, or enhanced habit responding, as proposed by the incentive-habit theory of addiction.
